# Real-time estimation of the remaining surgery duration for cataract surgery using deep convolutional neural networks and long short-term memory

**DOI:** 10.1186/s12911-023-02160-0

**Published:** 2023-05-04

**Authors:** Bowen Wang, Liangzhi Li, Yuta Nakashima, Ryo Kawasaki, Hajime Nagahara

**Affiliations:** 1 Institute for Datability Science (IDS), Osaka University, Suita, 565-0871 Japan; 2 Artificial Intelligence Center for Medical Research and Application, Osaka University Hospital, Suita, 565-0871 Japan; 3 Department of Vision Informatics, Graduate School of Medicine, Osaka University, Suita, 565-0871 Japan

**Keywords:** Surgery time, Cataract surgery, Long short-term memory

## Abstract

**Purpose:**

Estimating the surgery length has the potential to be utilized as skill assessment, surgical training, or efficient surgical facility utilization especially if it is done in real-time as a remaining surgery duration (RSD). Surgical length reflects a certain level of efficiency and mastery of the surgeon in a well-standardized surgery such as cataract surgery. In this paper, we design and develop a real-time RSD estimation method for cataract surgery that does not require manual labeling and is transferable with minimum fine-tuning.

**Methods:**

A regression method consisting of convolutional neural networks (CNNs) and long short-term memory (LSTM) is designed for RSD estimation. The model is firstly trained and evaluated for the single main surgeon with a large number of surgeries. Then, the fine-tuning strategy is used to transfer the model to the data of the other two surgeons. Mean Absolute Error (MAE in seconds) was used to evaluate the performance of the RSD estimation. The proposed method is compared with the naïve method which is based on the statistic of the historical data. A transferability experiment is also set to demonstrate the generalizability of the method.

**Result:**

The mean surgical time for the sample videos was 318.7 s (s) (standard deviation 83.4 s) for the main surgeon for the initial training. In our experiments, the lowest MAE of 19.4 s (equal to about 6.4% of the mean surgical time) is achieved by our best-trained model for the independent test data of the main target surgeon. It reduces the MAE by 35.5 s (-10.2%) compared to the naïve method. The fine-tuning strategy transfers the model trained for the main target to the data of other surgeons with only a small number of training data (20% of the pre-training). The MAEs for the other two surgeons are 28.3 s and 30.6 s with the fine-tuning model, which decreased by -8.1 s and -7.5 s than the Per-surgeon model (average declining of -7.8 s and 1.3% of video duration). External validation study with Cataract-101 outperformed 3 reported methods of TimeLSTM, RSDNet, and CataNet.

**Conclusion:**

An approach to build a pre-trained model for estimating RSD estimation based on a single surgeon and then transfer to other surgeons demonstrated both low prediction error and good transferability with minimum fine-tuning videos.

**Supplementary Information:**

The online version contains supplementary material available at 10.1186/s12911-023-02160-0.

## Introduction

Cataract surgery has become one of the most standardized procedures and visual acuity improves significantly with the surgery [1–4]. Establishing a standardized and quantifiable skill assessment for the training of surgeons is essential. There have been reports that the surgery length is associated with surgeons’ experience along with the case complexity and intraoperative complications [5–9], and this is also applicable to cataract surgery [10]. Shorter surgical length is associated with less risk of post-operative endophthalmitis [11]. Estimating the remaining surgery duration (RSD) has the potential to be utilized as information for education, training, or optimization of operating room scheduling, especially if it is done in real-time [12–19].

With the rapid development of convolutional neural networks (CNNs) [20–22], deep learning (DL) methods have achieved successful results in medical image analysis [23–33] including ophthalmic image classifications. The long short-term memory (LSTM) [34] has been proven to be useful in solving tasks such as estimating RSD [12, 13] for surgery. Cataract-101 [35] and related works [36] also successfully applied this technology for cataract surgery. However, there are two limitations still exist: (1) Annotation burden: Dataset like Cataract-101 needs a lot of annotation efforts. It requires the fine labeling of phases for each video, which is time costing. In fact, there are many unlabeled surgical video data that are protentional available to model training. For practical applications, it is important to efficiently utilize them with little annotation burden. (2) Transferability: Different hospitals may utilize different kinds of surgery equipment, and the camera type is also not the same. The model well trained in an environment setting may experience severe performance degradation in a new environment. Thus, the model transferability needs to be explored.

In this study, a CNN-based DL method that utilizes an LSTM structure is applied to realize the real-time RSD estimation for ophthalmic cataract surgery. We first compared our method to previous RSD methods in an open-source cataract surgery dataset Cataract-101. To explore the two limitations listed above, we aim to provide a method that only requires a few annotations for the data pre-processing step and can realize the RSD model training in an end-to-end manner. A fine-tuning strategy is also adopted to ensure that the trained model can be transferred among surgeons from different hospitals.

## Methods

### Data sets

**Fig. 1:**
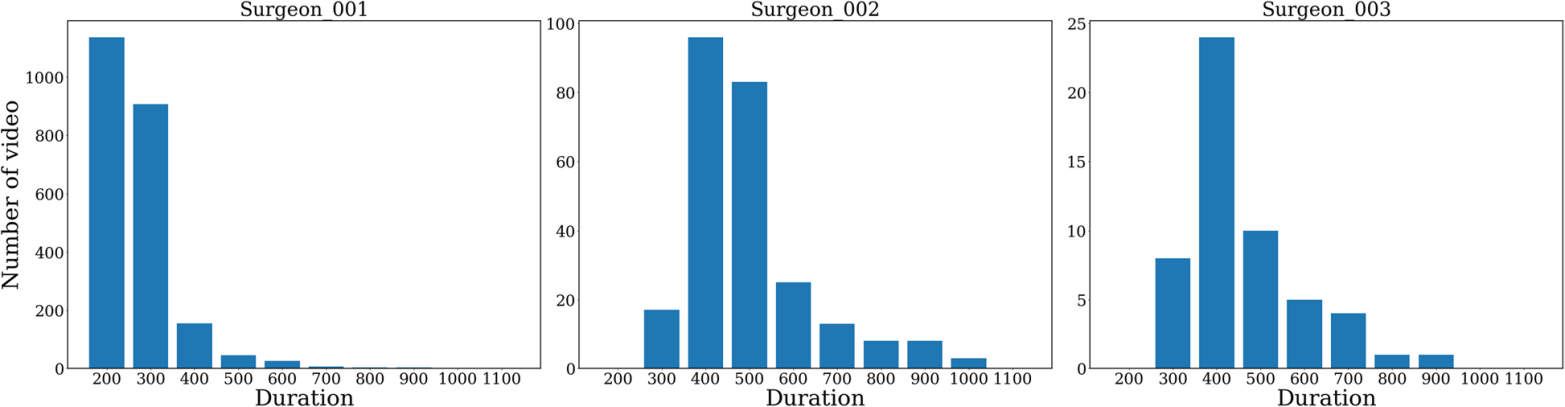
Duration histogram for each surgeon. The duration statistic of all three surgeons. All the videos are pre-processed with the TCN model. We set the interval of 100 s for demonstration and all the videos are decoded as 1 frame per second (FPS)

This study was approved by the institutional research board at the Osaka University Hospital. Our data contains 2,620 consecutive cataract surgery videos collected from 3 surgeons in 3 different hospitals. Cataract surgeries without unexpected complications were excluded, and typical surgical procedures were: (1) sclerocorneal incision/corneal incision, (2) replacing anterior chamber with viscoelastic agent, (3) Continuous Curvilinear Capsulorhexis (CCC), (4) hydrodissection, (5) phacoemulsification and aspiration, (6) lens cortex aspiration, (7) expanding capsule with viscoelastic agent, (8) intra-ocular lens insertion, (9) aspiration of the viscoelastic agent, (10) sclero-corneal/corneal would sealing. curvature continuous cystorvideos were recorded using CCD camera attached to the surgical microscope. Videos were captured as the high definition (HD) equivalent to 1280 × 720 pixels, and file formats were originally either.mov,.mp4, or.mts2/.mts. Each surgeon performed cataract surgeries independently in different hospitals. No videos were derived from the same surgery, and each video of the cataract surgery is independent. The background characteristics of the surgeon and surgical videos are shown in Table 1. All surgeons are well experienced in performing cataract surgeries and operate regularly, with their experience ranging between 5 to 25 years. Consecutive surgical videos conducting standard cataract surgeries were collected without specific selection. In Fig. 1, we show the distribution of surgery duration for each surgeon. All the videos are decoded as 1 frame per second (FPS). Thus, one frame represents the time of one second. We choose the surgeon 1 set as the main target as the surgeon has the most stable surgical time, shortest average time, and the largest number of surgical videos. Surgeon 1 set is used for the model pre-training. Other surgeon’s sets are applied for transferability evaluation.

**Table 1:** Statistic of our data set

Role of the dataset		Main model Pre-training	Transferability Evaluation	
**Hospital**		A	B	C
		**Surgeon 001**	**Surgeon 002**	**Surgeon 003**
**Video Number**		2310	256	54
**Mean ± standard deviation (s)**		318.7 ± 83.4	547.9 ± 146.3	515.5 ± 140.3
**Median (s)**		300.0	513.0	480.0
**Naïve MAE**	**Mean (s)**	54.9 ± 62.9	106.2 ± 100.6	104.6 ± 93.5
	**Mean (%)**	16.6 ± 14.5	19.1 ± 14.3	20.2 ± 16.1
	**Median (s)**	52.4 ± 67.7	102.3 ± 110.3	97.3 ± 107.2
	**Median (%)**	14.8 ± 13.8	17.1 ± 13.3	17.4 ± 15.5

The videos are decoded as 1FPS and pre-processed by the TCN model. The performance of the naïve method is calculated with mean and median (MAE in seconds and its percentage % of video duration)

*MAE* mean absolute error

### Method development

Before the estimation of RSD, we set a data pre-processing for all the videos in our data set to extract the exact surgical length. Previous works [12, 13, 17] directly apply the RSD estimation to the entire video. Typically, a recorded video contains a preparation phase and a post-operative phase including a procedure. We first used the temporal convolutional network [14] (TCN, structure shown in Supplement) to remove the preparation and post-operation phases that are not relevant to the true surgical length, and secure that the video starts with “sclero-corneal/corneal incision” and ends with “sclero-corneal/corneal wound sealing” procedures. For TCN, it is a three categories classification task to recognize the start frame, the end frame, and others. TCN will go through the whole video sequence and give each video frame a prediction. There is only one start and one end for a video.

**Fig. 2:**
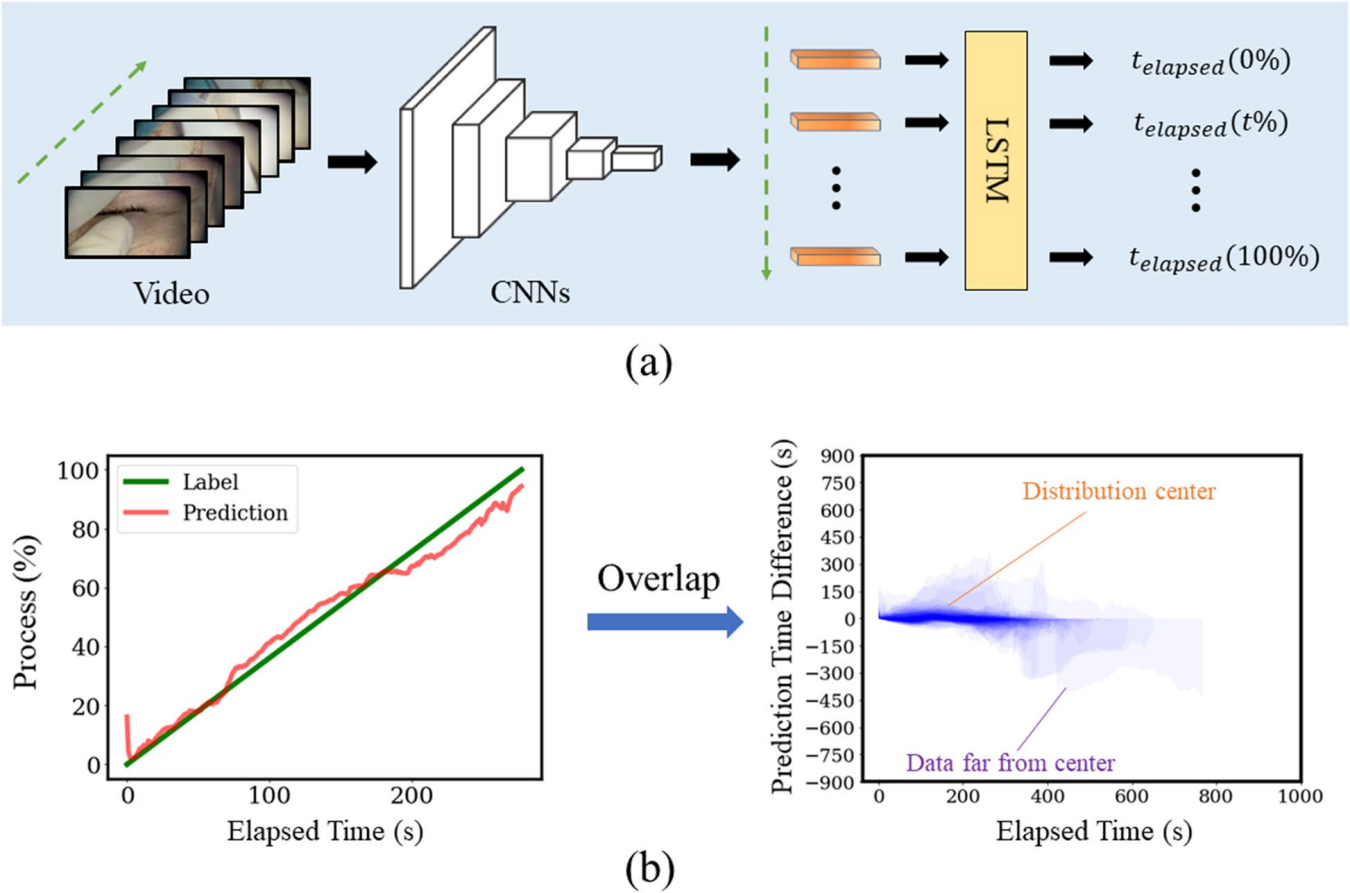
Structure of Proposed Method. **a** The proposed regression model which consists of CNNs and LSTM. CNNs are used to extract the features for each frame. LSTM will analyze the feature in time order and output the process (%) prediction for each elapsed time. **b** The figure on the left is the real-time prediction of a sample from surgeon 1’s test set. The horizontal axis is the true elapsed time (s), and the column axis is the progress (%) prediction of where the current time is during the whole video. The green line is the true label and the red line is the prediction. The figure on the right is the overlap map for observing the prediction error for the whole test set. The horizontal axis is the true elapsed time (s), and the column axis is the prediction error (s). The overlap map is drawn by overlapping the prediction error curve of all the test set samples of surgeon 1

To realize our main purpose of the real-time RSD estimation, we designed an end-to-end trainable regression model consisting of a CNNs feature extractor and a time series module LSTM (two-layers unidirectional). We show the overview of the model in Fig. 2-a. For one input video, the CNNs will first extract the features for each frame and obtain the feature vector ${f}_{t}$. The LSTM will then process the extracted features in a temporal order. For the loss calculation, each frame will have its own prediction with a proportional process value $\mathrm{s}$ ($\mathrm{s}=\mathrm{t}/\mathrm{T}$, where $\mathrm{T}$ is the maximum length of a video, where $\mathrm{t}$ is the elapsed time of the current frame). This value tells where the current frame is, as the percentage of the whole surgery. Since the elapsed time for the current processing frame is known, therefore, the length of the remaining time ${\widehat{y}}_{t}^{rsd}$ can be calculated. Another benefit of this method is that there is no need for manual annotation, as the ground truth can be automatically obtained by dividing the elapsed time of the current frame by the entire surgery duration. Since this is a linear regression task, we use the L1 loss as the loss function.

### Experiments setting and evaluation

We first compared our RSD method with previous works using an open-source dataset Cataract-101 [35]. This dataset contains 101 cataract surgery videos by four different surgeons with a resolution of 720 × 540 pixels acquired at 25 fps. Each video is annotated with 10 surgical phases and surgeon experience (senior or assistant surgeon). Our comparison refers to three existing RSD models as follows:

- (1) TimeLSTM [13]: A CNNs backbone is trained for phase classification and an LSTM is adopted for RSD prediction.
- (2) RSDNet [12]: It is a modified version of [13], predicting the progress and RSD.
- (3) CataNet [36]: This model is designed for the Cataract-101 dataset which uses both the image and elapsed time as CNNs’ input.

We mainly have two improvements for our RSD model: (1) All previous works train the backbone CNNs with a classification task and then fix its parameter during the continuous training of LSTM. We found that the frozen backbone restricts the feature extraction ability. Thus, in our setting, the whole model is trainable. (2) Instead of directly predicting the RSD as [], our model predicts the proportional process value $\mathrm{s}$, which enables the RSD prediction in a better form. Following the experiment setting of CataNet, we randomly split data into 81 for training and 20 for testing. fivefold cross-validation is adopted for model training. Video is down-sampled to 2.5FPS and the input size of each frame is 224 × 224. For a fair comparison, all the method adopts ResNet-50 [37] as the CNNs backbone and uses AdamW as an optimizer with a learning rate of 0.0001. The label of phase and surgeon experience are excluded during the training. The performance is evaluated by Mean Absolute Error (MAE = $\frac{1}{T}{\sum }_{t=0}^{T-1}\left|{\widehat{y}}_{t}^{rsd}-{y}_{t}^{rsd}\right|$), MAE-2 (MAE averaged over the last 2 min), and MAE-5 (MAE averaged over the last 5 min).

We applied a pre-training and fine-tuning strategy to the custom dataset for the evaluation of transferability. As shown in Table 4, we set surgeon 1 as the main training target and use fine-tuning to transfer the trained model (based on surgeon 1) to other surgeons’ samples. For surgeon 1, the training set, validation set, and test set are randomly split with a ratio of 80%, 10%, and 10%. For other surgeons, the ratio is 50%, 25%, and 25%. The model with the best performance in the validation set is saved during training. We adopt ResNet-18 [37] as the backbone CNNs and each input frame is resized to 224 × 224. We adopt a two-layers unidirectional LSTM with a hidden dimension of 512. The cell number of LSTM is decided by the video sequence length. The AdamW is adopted as an optimizer and the learning rate is set as 0.0001. We evaluated the RSD estimation by MAE. As a reference, in comparison to our method, we simply applied a Naïve approach [12]. This approach is defined as ${\widehat{y}}_{t}^{rsd}=max(0,{t}_{ref}-{t}_{el})$, where ${t}_{ref}$ is the referential duration derived from the dataset (statistic of the length of the videos, mean or median). ${t}_{el}$ is the time that has already passed at current time $\mathrm{t}$. We can simply calculate the MAE for all videos under the definition. This method requires no training but only relies on the statistic of the historical data of surgery length. The Naïve MAE for each surgeon is shown in Table 1.

All our experiments are implemented with a Tesla V100 32G GPU (Nvidia, CA, USA).

## Results

### Experiments on data pre-processing

Ophthalmology specialist (RK) provided the annotation for surgical procedures by the order of second for 100 videos for training the TCN for this preprocess. The result shows that the trained model shows very high performance (mean error < 2 s) in the independent test set. We further annotated a few videos (20 videos for each surgeon) for the videos of the rest three surgeons to test the transferability. The model still shows high performance (mean error < 5 s) for other surgeons’ videos that have never been accessed during training. As shown in Table 2, the ACC and AUC represent the prediction accuracy of labeled start and end frames. It is a three-classes classification problem (start frame, end frame, and others). ACC is the classification performance for all three categories. AUC@start and AUC@end are used to evaluate the recognition of start and end frames respectively. We can observe that the TCN model achieved high performance. Even for the worst result from surgeon 3, the time difference is smaller than 5 s.

**Table 2:** Prediction accuracy of TCN in ours dataset

	Acc	AUC@start	AUC@end
Surgeon 1	0.991	1.000	0.997
Surgeon 2	0.970	0.997	0.962
Surgeon 3	0.962	0.995	0.957

### Experiments on cataract-101 dataset

In Table 3, we compared our RSD model with previous methods on the Cataract-101 dataset. The results are evaluated by MAE for seniors’ videos, assistants’ videos, and all the videos. We can observe that our method outperforms previous works in almost all the settings. For the most important setting of MAE for all the videos, our method decreases the MAE of 24.8 s, 12.8 s, and 4.9 s than TimeLSTM, RSDNet, and CataNet respectively. Depending on the surgeon's experience, Cataract-101 has two types of surgical videos and there is an obvious prediction accuracy gap between senior and assistant. Our method reduces this gap to 4.1 s, where this gap was 49.2 s, 32.5 s, and 12.8 s for TimeLSTM, RSDNet, and CataNet, respectively.

**Table 3:** Comparison to previous methods on Cataract-101

	Experience	TimeLSTM	RSDNet	CataNet	Ours
	All	103.2 ± 52.2	99.2 ± 47.3	92.2 ± 40.8	**88.3 ± 35.5**
MAE-5	Senior	133.7 ± 56.8	124.2 ± 51.7	98.7 ± 44.5	**91.6 ± 41.2**
	Assistant	72.6 ± 18.5	76.1 ± 20.4	85.6 ± 29.1	**84.9 ± 22.8**
	All	92.9 ± 27.6	86.0 ± 27.2	78.6 ± 22.6	**76.9 ± 22.1**
MAE-2	Senior	100.2 ± 24.7	95.8 ± 28.4	**80.7 ± 19.2**	82.2 ± 24.8
	Assistant	85.5 ± 22.7	76.1 ± 25.0	75.8 ± 21.0	**71.5 ± 18.4**
	All	115.6 ± 43.2	103.6 ± 45.9	95.7 ± 40.5	**90.8 ± 37.9**
MAE	Senior	140.2 ± 45.1	119.8 ± 48.6	102.1 ± 38.8	**92.8 ± 32.1**
	Assistant	91.0 ± 39.3	87.3 ± 40.1	89.3 ± 42.3	**88.7 ± 39.3**

The MAE (mean ± std, in seconds) is shown for entire videos, MAE-2 for last two minutes and MAE-5 for last five minutes

We also implement an ablation study to evaluate the impact of our two improvements on the RSD model. As shown in Table 4, there are 3 comparisons: (i) “Base” represents the results of proposed RSD model, (ii) “Fix” represents the model trained with backbone fixed, and (iii) “RSD” represents the model is trained to directly predict RSD. After fixing the backbone during training, the mean of MAE for almost all settings is only slightly increased (about 1 s). However, the variance shows an obvious rising (about 7 s). It implies that training the entire model can contribute to a more robust RSD prediction. We can also observe an obvious improvement when using proportional process value $\mathrm{s}$ for predicting RSD. It can reduce the MAE by 5.6 s. In general, the experimental results on Cataract-101 demonstrate the superiority of our RSD method.

**Table 4:** Ablation for RSD prediction on cataract-101

	Experience	(i) Base	(ii) Fix	(iii) RSD
	All	88.3 ± 35.5	90.4 ± 40.1	94.9 ± 34.2
MAE-5	Senior	91.6 ± 41.2	95.5 ± 46.0	98.2 ± 40.9
	Assistant	84.9 ± 22.8	85.3 ± 23.4	91.5 ± 21.1
	All	76.9 ± 22.1	78.9 ± 25.3	83.7 ± 24.3
MAE-2	Senior	82.2 ± 24.8	84.7 ± 29.4	86.8 ± 24.6
	Assistant	71.5 ± 18.4	73.0 ± 21.6	80.6 ± 19.0
	All	90.8 ± 37.9	91.2 ± 42.8	96.4 ± 39.5
MAE	Senior	92.8 ± 32.1	97.9 ± 36.2	99.6 ± 30.4
	Assistant	88.7 ± 39.3	84.5 ± 45.8	93.1 ± 41.7

### Experiments on the custom dataset

For the continuous part, we analyzed the experiment results on our custom dataset. In Table 5, we show the experimental results of RSD estimation for the proposed method in the test set. 86.6% of surgeon 1’s videos have the surgical length mostly ranging between 200 and 300 s, and only 1.7% of videos are with durations over 400 s. As shown in Table 1, the mean and median of all the videos of surgeon 1 are 318.7 s and 300.0 s respectively. The MAEs of the two naïve settings are 54.9 ± 62.9 s and 52.4 ± 67.7 s, which show no obvious difference between each other. The proposed method has much better performance than the naïve method (reduces the MAE by 35.5 s and 33.0 s (10.2% and 8.4%) compared to the mean and median naïve method, respectively), and the MAE for the proposed method is 19.4 s, with a variance of 24.9 s. The MAE in prediction is only about 6.4% of the video duration. Since our model can serve as a real-time regression prediction, for each second, there is the prediction for the corresponding process value. In Fig. 2-b left, we demonstrate the prediction of one sample from surgeon 1’s test set. We can observe that the prediction curve (red line) is around the true process slash (green line). The vertical distance between two lines is the prediction error. The prediction of one sample will get a MAE of 0 when the prediction curve is matched up with the true process slash.

**Table 5:** Results of the RSD estimation

Data	Models	MAE (s)	MAE (%)	Difference in the MAE (s)	*p*-value ^*^
Surgeon 001	Per-surgeon model	19.4 ± 24.9	6.4 ± 4.6	-	-
Surgeon 002	Per-surgeon model	36.4 ± 16.0	5.8 ± 2.9	Reference	-
	Pre-trained with Surgeon 1 model	94.5 ± 48.3	18.1 ± 7.6	+ 58.1 s	< 0.001
	Fine-tuned model (number for fine-tuning = 128 videos)	28.3 ± 19.0	5.2 ± 2.8	-8.1 s	< 0.001
Surgeon 003	Per-surgeon model	38.1 ± 18.5	9.3 ± 6.8	Reference	-
	Pre-trained with Surgeon 1 model	87.4 ± 39.9	17.2 ± 8.0	+ 49.3 s	< 0.001
	Fine-tuned model (number for fine-tuning = 27 videos)	30.6 ± 15.3	7.9 ± 4.0	-7.5 s	0.003

Per-surgeon model: a model without fine-tuning using per-surgeon’s data for training. Pre-trained model: a model only uses the pre-trained parameter from surgeon 1 to surgeons 2 and 3. Fine-tuned model: a model using the pre-trained parameter from surgeon 1 and fine-tuned with the training data. MAE: mean absolute error

^*^ t-test compared to the per-surgeon model

In Fig. 2-b right, we also show an overlap map for observing the prediction error for the whole test set. The horizontal axis is the elapsed time of the surgery and the column axis is the prediction error which can be both positive and negative. The prediction error results of all the videos in the test set are overlapped on this figure. There is a high-density part around the horizontal axis (time < 400 s), with an error range of -15 s ~ 15 s. We can find that the prediction error is small for the videos near the center (200 s < time < 300 s) of the duration distribution, while the videos far from the center range may have a large prediction error. This is consistent with the data statistic in Table 1 and Fig. 1. We also draw the box plots by every 50 s in the supplement. It obviously shows the prediction error of each elapsed time. This also proves that the model has better prediction results around the center of the distribution.

**Fig. 3:**
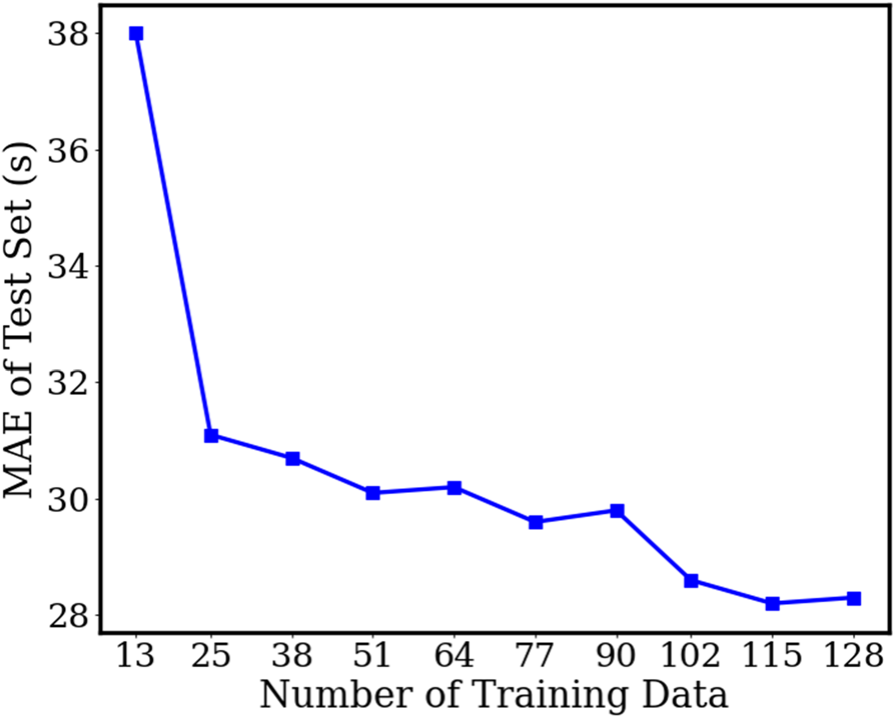
The experiment for fine-tuning data quantity evaluation. The horizontal axis is the number of training data used for fine-tuning the model. We take those numbers by every 10% of the training data from surgeon 2. The column axis is the MAE estimation of surgeon 2’s test set

**Fig. 4:**
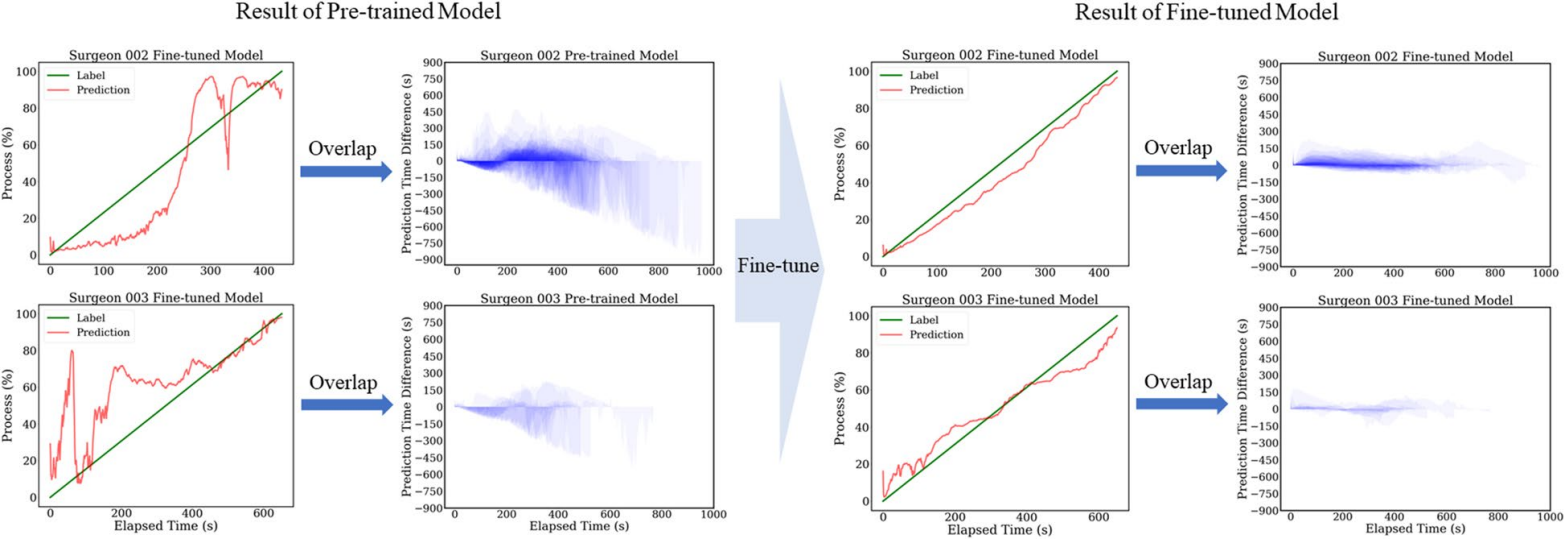
The experiments result for surgeon 2 and 3. The results of the pre-trained model and fine-tuned model are shown on the left and right, respectively. From left to right, we can observe the improvement of the real-time prediction samples and the overlap maps. All the results are calculated by the test set of each surgeon

**Fig. 5:**
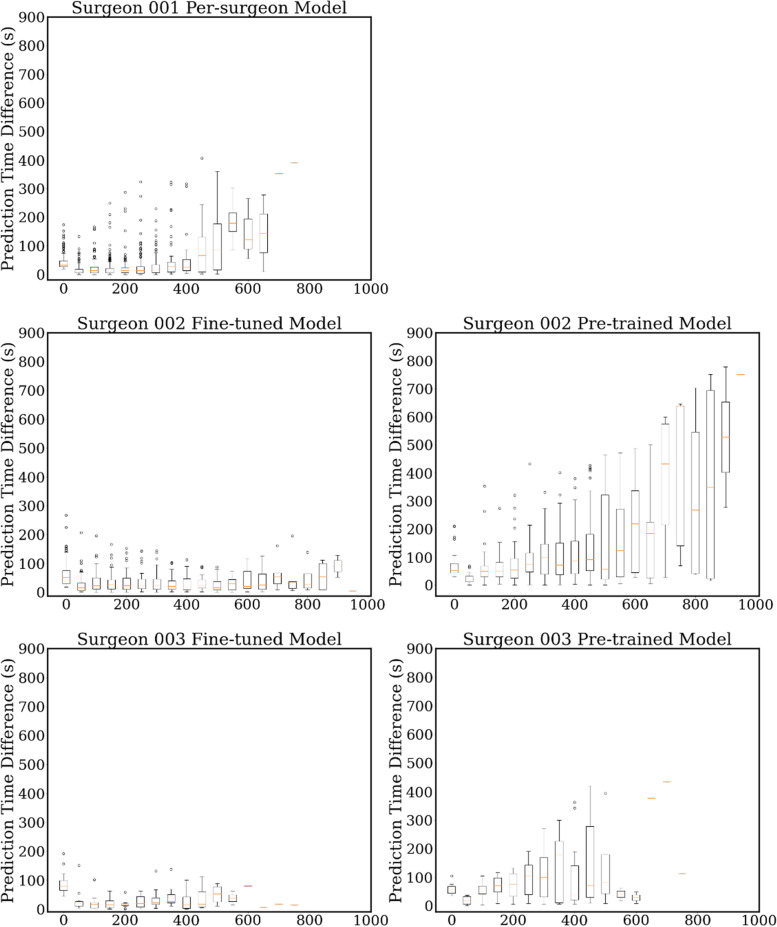
The demonstration of plot maps. This is the extension of the overlap map shown in Figs. 2 (b) and 4. The horizontal axis is the elapsed time (s), and the column axis is the prediction error (s). For each surgeon, the box plots map is drawn by every 50 s on the horizontal axis

Compared to surgeon 1’s duration distribution, the distributions of surgeon 2 and 3 are sparser. As shown in Table 1, they have a larger mean duration and variance, which further worsen the performance of the naïve method. The MAE results of both naïve settings for those surgeons are almost all over 100 s. This is a large prediction error which reflects that the naive method cannot well deal with the data that is with a sparse distribution. We directly applied the model trained with the data of surgeon 1 to the data of other surgeons. We further implement an experiment on surgeon 2 for verifying how much data are necessary for fine-tuning the model. The results (Table 5, shown as the Pre-trained model) are better than the naïve but are still not satisfactory. This is possibly due to the different characteristics (such as surgeon skills, surgery environments, etc.) of each surgeon’s data, especially the surgeons belonging to different hospitals. The fine-tuning strategy can obviously improve the model performance. Figure 3 shows the number of used training data (every 10% of the whole training data) in the horizontal axis and the column axis is the MAE evaluation results of the test set for surgeon 2. We first randomly separated the training set into ten subsets (from number 1 to 10) of equal size. We then gradually add the subset into the model train by the number order. Thus, the test results are only related to the training size. We can find that the MAE only slightly increased (about 3 s) when setting the ratio of training data from 20% (25 videos) to 100% (128 videos). This supports the fine-tuning strategy for the surgeon 3, which has relatively small number of videos. To tune the model, only a small number of data (27 as the minimum in our case) for each surgeon are needed. With the fine-tuning strategy, the MAEs for these two surgeons are 28.3 s and 30.6 s, which decreased by -8.1 s and -7.5 s than the Per-surgeon model (average declining of 7.8 s and 1.3% of video duration). In Fig. 4, shown from left to right, the fine-tuned model has much better performance than the pre-trained model. In addition, the results of the overlap map show similar trends with surgeon 1. The samples near the distribution center have low prediction errors, while the predictions for other videos are usually with higher errors. We also show the extension plot map of each surgeon in Fig. 5.

## Discussions

In this paper, we designed an end-to-end trainable regression model to realize a real-time estimation of the remaining surgical duration for cataract surgery. In an open-source dataset Cataract-101, our RSD method outperforms the best competitor by 4.9 s. We also released a custom dataset with 2620 surgery videos to explore the limitation of previous works. The experimental results prove that the proposed method has a low prediction error (MAE of 19.4 s) and can be easily transferred among different surgeons with minimum fine-tuning. Although cataract surgery is one of the most standardized surgeries, surgical time for each case is highly variable depending on the surgeons’ experience along with the case complexity [7]. The use-case of our proposed RSD estimation has mainly three clinical benefits. Firstly, shorter surgical time is associated with better surgical outcomes and less risk of post-operative endophthalmitis [8]. The proposed RSD estimation model can be utilized as a tool to record and monitor surgical time. Secondly, the estimation of surgical time can be considered as an average surgical time based on past surgeries. Thus, it acts as a benchmark surgical time so that one can evaluate the actual surgical time to identify which procedure can be improved. As shown in Fig. 4, there are differences against the estimation in whether the actual time is faster or slower than the estimation which is considered as an averaged procedure time. Comparing the actual time to the estimated time by procedures can suggest which procedure has reached the average time, and which procedure has not. Having this information in real-time will help a supervisor to understand the progress by bench marking their skills. Thirdly, real-time estimation has the most potential for better efficiency in utilizing resources of surgical staff and surgical facilities such as operating rooms and instruments essential to provide sustainable medical services. Optimizing surgical facilities can contribute to enhancing patient experiences by minimizing waiting time and allocating training time for trainee surgeons.

There are variations in the procedure order, length, and instruments used. Therefore, rather than having a single model, we hypothesized that the highly individualized model by light fine-tuning for each surgeon performs better. We adopted a “pre-training and minimum fine-tuning” strategy, and we achieved good estimation without further data labeling work and efficient process flow. Our strategy can be reproduced by starting from a pre-training base model, and then using very lightweight fine-tuning of < 50 videos for each specified surgeon to realize the transfer of the model parameter. Especially for the videos near the duration distribution center, the prediction error is small. However, for the videos with uncommon durations (e.g., too long or short), the model may have large prediction errors. This can be caused by the data imbalance, i.e., the model will be adjusted to better fit into most samples during the training process while ignoring some uncommon cases.

We have several observations in fine-tuning strategy that can perform well with small samples, still there were better outcome with more samples were available. For example, surgeon 2 has video samples of *n* = 128 and the fine-tuning results are better than those of surgeon 3 who has less number for fine-tuning (*n* = 27). If we aim for the model to achieve high accuracy, it can be fine-tuned with at least 100 videos from each surgeon. We consider it is still a reasonable number for additional annotation workload, as this model only requires the starting frame and the ending frame for training the model.

The limitations of this study should be stated. Firstly, more variation in the dataset will contribute to the analysis of robust RSD estimation for cataract surgery videos. Our dataset has 2,620 ophthalmic cataract surgery videos of four surgeons collected from three different hospitals with relatively experienced surgical skills. For transferability experiments, we only have three surgeons. We will add more surgeons for future studies with various experiences, especially less experienced surgeons. Additional experimental subjects will increase the robustness of the proposed method. Secondly, we only used ResNet-18 as the feature extractor in our model construction. Generally, a larger backbone (e.g. ResNet-50) will enhance the fitting ability of the model. However, we train the model in an end-to-end manner and the real-time prediction task maximally requires the whole video as the input. The GPU memory limited the selection of the backbone.

## Conclusions

In this paper, we designed an end-to-end trainable regression model to realize a real-time estimation of the remaining surgical duration for cataract surgery. In an open-source dataset Cataract-101, our RSD method outperforms the best competitor by 4.9 s. We also released a custom dataset with 2620 surgery videos to explore the limitation of previous works. The experimental results prove that the proposed method has a low prediction error (MAE of 19.4 s) and can be easily transferred among different surgeons with minimum fine-tuning. We believe this method can contribute to expanding the potential of utilizing real-time RSD information for surgical training and optimization of surgical facilities and resources.

## Supplementary Information

**Additional file 1.**

## Data Availability

All our data and source codes are available by contacting the corresponding author or first author. Due to the privacy issue, our dataset can be distributed after joining our research group.
