# Supplementary material for: Real-time estimation of the remaining surgery duration for cataract surgery using deep convolutional neural networks and long short-term memory
Source: BMC Med Inform Decis Mak. 2023 May 4;23:80. doi: 10.1186/s12911-023-02160-0 (PMC10161556; doi:10.1186/s12911-023-02160-0)

**Supplement**

**Fig S1. Model Structure of TCN.**

The TCN model takes the input video vectors stream $x$ as input. The x of each time skip t is the feature vector extracted from the CNNs backbone. (ResNet-18 is applied in our experiments.) For each hidden layer, TCN applied the 1-D dilated convolution to scan the temporal dimension and output the final label prediction for each time skip. For our pre-processing task, the prediction of y is whether the current frame is the start frame or the end frame. Thus, it is a classification task for each frame with three categories start, end, and others. Please refer to the original paper “An empirical evaluation of generic convolutional and recurrent networks for sequence modeling” for details.


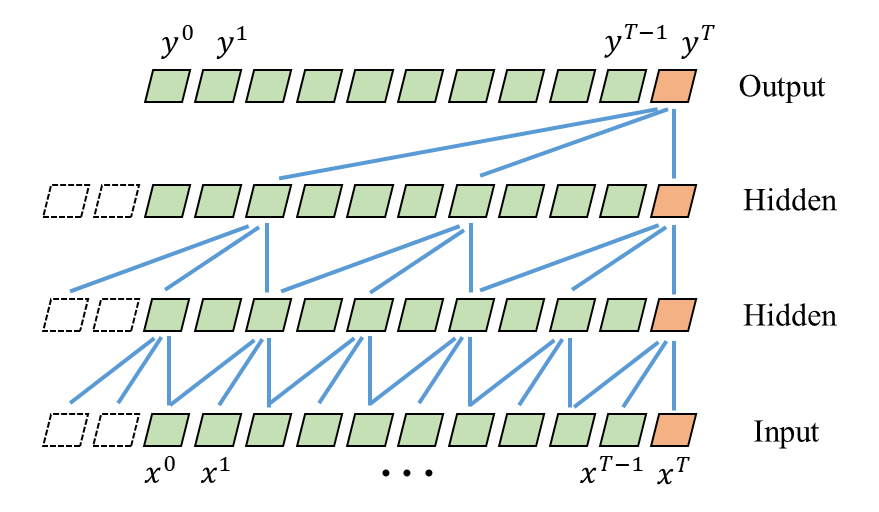

Supplement: Supplementary file 1 — Additional file 1. [file 12911_2023_2160_MOESM1_ESM.docx]
